# Supplementary material for: Percutaneous pericardial catheter drainage for symptomatic uremic pericardial effusions with narrow safety margins
Source: PLoS One. 2022 Oct 31;17(10):e0276498. doi: 10.1371/journal.pone.0276498 (PMC9621416; doi:10.1371/journal.pone.0276498)
Supplement: Attachment — Submitted filename: Response to Reviewers.doc [file pone.0276498.s001.doc]

**Dear Dr Arturo Cesaro**

 We have received your decision letter regarding our manuscript PONE-D-21-26425, entitled "Percutaneous Pericardial Catheter Drainage for Symptomatic Uremic Pericardial Effusions with Narrow Safety Margins". We were happy to learn that our manuscript will be reconsidered for publication pending the completion of revisions.
 We have changed the manuscript as much as possible according to the reviewer’s comments. We wish to thank you and the reviewers for your valuable comments and helpful suggestions which contributed significantly to the revision of our manuscript. We ensured that our manuscript met PLOS ONE’s style requirements and Full ethics statement was included in the ‘Methods’ section. We are also sending our responses to the comments of the reviewers in a point-by-point fashion.

We updated the Manuscript and Tables by increasing the number of the cases from 9 to 13. We ensure that our manuscript met PLOS ONE's style requirements, including those for file naming. We also ensure that our manuscript included our full ethics statement in the ‘Methods’ section of our manuscript file.

**Response to First reviewer**

**REV 1:** *Authors describe a case series of patients with symptomatic large uremic pericardial effusion with narrow safety margins for pericardiocentesis with subxyphoid access. Patients underwent pericardiocentesis under echocardiographic and fluoroscopic guidance. The manuscript is written in clear standard English. It is a retrospective description of the cases.*

*There are some major revisions required:*

*1) to provide echocardiographic data on the absence/presence of tamponade/pre-tamponade diagnostic signs;*

**☞ We added the following into PATIENTS (Page 5, Lines 88~95) and Table 2. – “We provided the echocardiographic data on the absence/presence of tamponade/pre-tamponade diagnostic signs such as “respiratory variation of mitral inflow or diastolic collapse of right atrium or right ventricle in addition to inferior vena cava plethora in Table 2. Signs of pericardial tamponade were found in 9 cases; definitely in 7 and equivocally in 2. Eight cases had large effusion on the echocardiographic findings (circumferential effusion with the echo-free space greater than 20 mm at the widest point. All six patients who had equivocal or no evidence of tamponade had echocardiographically large pericardial effusion. We could not find the results of paradoxical pulse.”**

*2) to better explain indications to procede to pericardiocentesis even in the absence of tamponade or of the demonstration of resistance to treatment (why intensive dialysis wasn't the preferred first line strategy in all the cases? - Am J Med Sci. 2003 Apr;325(4):228-36);*

**☞ We agree with the Reviewer’s comment. We should have written the reason why we have performed this aggressive intervention. We added the following in the INTRODUCTION (Page 3, Lines 44~57) and Table 1.**

**“Although intensive dialysis is the first line strategy in uremic patients with pericardial effusion [2], previously we lost two patients with symptomatic pericardial effusion without signs of tamponade with narrow safety margin in spite of intensive dialysis (Table 1). In Case 1 who were on continuous ambulatory peritoneal dialysis (CAPD) for 7 years, the patient died of pericardial tamponade in spite of continuing CAPD 4 times a day plus 5 times of hemodialysis during 13 days. Intensification of hemodialysis (5–7 days per week) is only effective approximately 50% of the time in dialysis-associated pericarditis (dialysis over 8 weeks) [3]. Although uremic pericarditis (prior to or within 8 weeks of initiation of dialysis) responds extremely well to initiation of dialysis [3], our Case 2 died of pericardial tamponade in spite of hemodialysis 7 times during 10 days. In addition, there is no guideline how long we should do this “intensive dialysis”. Thereafter, we actively proceed to pericardiocentesis in patients with symptomatic uremic pericardial effusion without safety margin even in the absence of tamponade. Another reason for active pericardiocentesis is early differential diagnosis of other causes of pericardial effusion, such as malignancy, bacterial or tuberculosis.”**

*3) to explain why it was not considered to use approach other than subxiphoid, since with echocardiographic guidance it is possible to perform safely apical and parasternal approaches to reach the maximum amount of fluid to overcome the absence of standard safety margins in the subxiphoid approach;*

**☞ Indeed, in this study, the PCD procedures were performed by an interventional radiologist familiar with the subxiphoid approach. Apical or parasternal approaches are unfamiliar due to the lack of sufficient knowledge of echocardiography. And the subxiphoid approach was considered safer.**

*4) to remove references to UpToDate website and specifically cite papers that support the statements.*

**☞ We removed UpToDate website references and specifically cited articles or books.**

**Response to Second reviewer**

I read your article with great interest. Pericardial effusion can occur in many metabolic and oncological diseases. It is a condition with a high risk of recurrence if not treated with an appropriate approach. We have interventional and surgical options for intervention. There are many studies comparing these two methods in terms of safety and effectiveness. I would like to explain my criticisms about the study you have done, item by item, in the continuation of the article:
**REV 2-1:** *In the study, you have presented the results of seven patients. It means that two patients had to be re-intervented with PCD. In addition to that, the 8th patient had undergone surgery one month after PCD. Actually, these outcomes indicate that PCD was a permanent solution for only four patients. In your article, you mentioned that the cardiologists had rejected the PCD because of the narrow safety margin. Cardiologists and Cardiothoracic Surgeons are the professionals who manage the process of heart-related medical conditions. Cardiologists might reject the PCD because of the high risk but they ought to suggest a better and less risky way to fix the problem. In your study, you did not mention what their suggestions were instead of PCD. Every high-risk intervention could be performed successfully by some talented medical associates. Having abilities to perform high-risk interventions is not a scientific justification to do it. Patients' safety and permanency of the therapy must be the main goals of every healthcare professional. In the guideline, the recommendation for pericardial effusion with a known etiology is primarily to treat the underlying disease. Pericardial effusion is expected to decrease in this way. It was stated in your study that 3 patients did not receive any renal replacement therapy prior to PCD. One of these patients had also anasarca. Why didn’t you perform dialysis for these three patients primarily?*
**☞ We agree with the Reviewer’s comment. This comment is similar to reviewer 1's comment. Cardiologists who are not proficient in PCD tend to be somewhat reluctant in challenging drainage. We added 4 cases (4 patients) after we submitted the original manuscript at August 2021. Finally we report 13 cases.**

**We described the reason why we have performed this aggressive intervention by adding the following in the INTRODUCTION (Page 3, Lines 44~57) and Table 1.**

**“Although intensive dialysis is the first line strategy in uremic patients with pericardial effusion [2], previously we lost two patients with symptomatic pericardial effusion without signs of tamponade with narrow safety margin in spite of intensive dialysis (Table 1). In Case 1 who were on continuous ambulatory peritoneal dialysis (CAPD) for 7 years, the patient died of pericardial tamponade in spite of continuing CAPD 4 times a day plus 5 times of hemodialysis during 13 days. Intensification of hemodialysis (5–7 days per week) is only effective approximately 50% of the time in dialysis-associated pericarditis (dialysis over 8 weeks) [3]. Although uremic pericarditis (prior to or within 8 weeks of initiation of dialysis) responds extremely well to initiation of dialysis [3], our Case 2 died of pericardial tamponade in spite of hemodialysis 7 times during 10 days. In addition, there is no guideline how long we should do this “intensive dialysis”. Thereafter, we actively proceed to pericardiocentesis in patients with symptomatic uremic pericardial effusion without safety margin even in the absence of tamponade. Another reason for active pericardiocentesis is early differential diagnosis of other causes of pericardial effusion, such as malignancy, bacterial or tuberculosis.”**

**REV 2-2:** *The guideline recommends intervention for chronic (>3 months) and large pericardial effusions. There are only two patients who showed signs of pericardial tamponade(IVC plethora, 2 and 9). Dyspnea, hemoptysis, and orthopnea occur in pulmonary edema due to volume overload. These are not specific findings of pericardial tamponade. Were there other findings for pericardial tamponade, such as an inspiratory change in blood pressure?*

**☞ We added the following into the PATIENTS (Page 5, Lines 88~95) and Table 2 – “We provided the echocardiographic data on the absence/presence of tamponade/pre-tamponade diagnostic signs such as “respiratory variation of mitral inflow or diastolic collapse of right atrium or right ventricle in addition to inferior vena cava plethora in Table 2. Signs of pericardial tamponade were found in 9 cases; definitely in 7 and equivocally in 2. Eight cases had large effusion on the echocardiographic findings (circumferential effusion with the echo-free space greater than 20 mm at the widest point. All six patients who had equivocal or no evidence of tamponade had echocardiographically large pericardial effusion. We could not find the results of paradoxical pulse.”**

**REV 2-3:** *How long was the pericardial effusion followed in these patients? How long was the medical treatment given? Was the intervention planned as soon as pericardial effusion was detected?*

**☞ We added the following into the PATIENTS (Page 5, Lines 99~100) and Table 2. – “We provided the data about the time from detection of pericardial effusion to PCD, which was median 2 days (range, same day~8 months). PCD was performed within 8 days in 10 out of 13 cases.”.**

**REV 2-4:** *In echocardiographic measurements, the distribution of pericardial effusion is highly variable in a few cases. Was further examination(CT or CMR) performed considering the possibility of loculations? Because the presence of loculation further reduces the chance of success of percutaneous interventions.*
**☞ We added the following into the PATIENTS (Page 5, Lines 96~98) and Table 2.- “Chest CT was performed in 9 out of 13 cases to make sure encircling nature of pericardial effusion, because the presence of loculation further reduces the chance of success of percutaneous interventions.”.**

**REV 2-5:** *Finally, I think that a 9-case study is not sufficient to recommend a high-risk intervention as "safely feasible". If you could increase the number of cases and data, it might be a considerable study.*
**☞ We agree with the Reviewer’s opinion. We added 4 cases after we submitted the original manuscript at 2021 August. Finally we report 13 cases. Manuscript and tables were updated as the number of patients increased.**

Thank you very much
