# Supplementary material for: Percutaneous pericardial catheter drainage for symptomatic uremic pericardial effusions with narrow safety margins
Source: PLoS One. 2022 Oct 31;17(10):e0276498. doi: 10.1371/journal.pone.0276498 (PMC9621416; doi:10.1371/journal.pone.0276498)
Supplement: Attachment — Submitted filename: Response to Reviewers.doc [file pone.0276498.s002.doc]

**Dear Dr Wisit Kaewput**

 We have received your decision letter regarding our manuscript PONE-D-21-26425R1, entitled "Percutaneous Pericardial Catheter Drainage for Symptomatic Uremic Pericardial Effusions with Narrow Safety Margins". We were happy to learn that our manuscript will be reconsidered for publication pending the completion of revisions.
 We have changed the manuscript as much as possible according to the reviewer’s comments. We wish to thank you and the reviewers for your valuable comments and helpful suggestions which contributed significantly to the revision of our manuscript. We ensured that our manuscript met PLOS ONE’s style requirements and Full ethics statement was included in the ‘Methods’ section. We are also sending our responses to the comments of the reviewers in a point-by-point fashion.

We ensure that our manuscript met PLOS ONE's style requirements, including those for file naming. We also ensure that our manuscript included our full ethics statement in the ‘Methods’ section of our manuscript file.

**Response to Second reviewer**

**REV 2-1:** *In your revised paper, I couldn't find the answer to "what was the cardiologists' suggestion instead of the PCD?". In my opinion, surgery(pericardial window) would have been a safer and more permanent treatment option for all the patients in your study. I think that the cardiologists suggested the surgery instead of PCD.*

**☞ We should have written the cardiologists' suggestion. We added the following into Discussion section (Page 8, Lines 160~164).**

**“Cardiologists have suggested that more observation or surgical treatment should be considered as PCD is technically difficult. However, there were practical problems such as a surgeon's tight schedule to consider surgical treatment, and there were cases where surgery could not be considered due to the rapid deterioration of the patient's situation. Therefore, it is considered as a didactic article that the indication of percutaneous pericardial catheter drainage can be broadened.”**

**REV 2-2:** *Patient 1 had a large pericardial effusion upon admission (Posterior of LV, 37mm). Surgery would be the option for treatment. The PCD is not a feasible method for this case.*

*Patient 2 didn't have pericardial effusion that needed an aggressive intervention.*

*In pre-mortem echo findings, you have mentioned massive pericardial effusion. What are your measurements?*

*These two patients are not similar cases. They can't be a medical basis for your study.*

**☞ There is an interval of 13 or 6 days between initial echocardiography and death. Bedside echocardiography just before death did not show any detailed measurements, but there was a lot of pericardial effusion. In case of patient 2, the amount of pericardial effusion was not large on the initial echocardiography, but clinical symptoms such as dyspnea and orthostatic respiration required treatment.**

**We have added the following sentence in Introduction section (Page 3, line 54, 55) for clarity.**

**“The reason for mentioning these two patients is to show that untreated symptomatic pericardial effusion with a narrow safety margin can be fatal.”**

**REV 2-3:** *Could you please mention the post-interventional echo findings in Table 2?*

**☞ We did not perform post-interventional echo in most cases, because there were large amounts of drainage and dramatic improvements in symptoms and chest radiographs. We added the following in the Materials and Methods section (Page 6, Lines 103~104).**

**“Echocardiography was not performed after PCD because there was a large amount of drainage and there was a dramatic improvement in symptoms and chest radiographs.”.**

**Response to Third reviewer**

**REV 3-1:** *Authors have addressed the reviewers' comments a properly. I have one minor change to suggest - the date format in table 1 and table 2 (yearmonthdate) are not in universal format. In fact, presenting the exact date in the manuscript might not be appropriate because this is patient identifiable information.*

**☞ We agree with you. ‘Time from initial echo to death’ information is sufficient. We have deleted the date information for ‘Date of initial echo’ and ‘Date of death’.**

Thank you very much
